# Supplementary material for: Association of Chronic Periodontitis with Migraine in a Korean Adult Population: A Nationwide Nested Case-Control Study
Source: Healthcare (Basel). 2025 Aug 26;13(17):2123. doi: 10.3390/healthcare13172123 (PMC12428593; doi:10.3390/healthcare13172123)
Supplement: Supplementary file 1 [file healthcare-13-02123-s001.zip › Table S1 (Migraine) - d.pdf]

Table S1. Subgroup analyses of crude and adjusted odds ratios according to age, sex, income, and region of residence

Characteristics	No. of case	No. of control	Odds ratios for migraine (95% confidence interval)					
	(exposure/total, %)	(exposure/total, %)	Crude†	P-value	Model 1†‡	P-value	Model 2†§	P-value
Age <60 years old (n = 98,365)								
CP ≥1 (1 year)	3964/19,673 (20.2%)	14,859/78,692 (18.9%)	1.08 (1.04-1.13)	<0.001*	1.09 (1.05-1.14)	<0.001*	1.09 (1.05-1.14)	<0.001*
CP ≥2 (1 year)	1843/19,673 (9.4%)	7119/78,692 (9.1%)	1.04 (0.99-1.10)	0.157	1.05 (0.99-1.10)	0.104	1.05 (0.99-1.11)	0.093
CP ≥3 (1 year)	1008/19,673 (5.1%)	3966/78,692 (5.0%)	1.02 (0.95-1.09)	0.63	1.03 (0.95-1.10)	0.491	1.03 (0.96-1.10)	0.464
CP ≥1 (2 years)	6278/19,673 (31.9%)	23,475/78,692 (29.8%)	1.10 (1.07-1.14)	<0.001*	1.11 (1.08-1.15)	<0.001*	1.11 (1.08-1.15)	<0.001*
Age ≥60 years old (n = 118,430)								
CP ≥1 (1 year)	5822/23,686 (24.6%)	21,520/94,744 (22.7%)	1.11 (1.07-1.15)	<0.001*	1.12 (1.08-1.15)	<0.001*	1.11 (1.08-1.15)	<0.001*
CP ≥2 (1 year)	2873/23,686 (12.1%)	11,030/94,744 (11.6%)	1.05 (1.00-1.10)	0.035*	1.05 (1.01-1.10)	0.020*	1.05 (1.01-1.10)	0.026*
CP ≥3 (1 year)	1576/23,686 (6.7%)	6276/94,744 (6.6%)	1.00 (0.95-1.06)	0.87	1.01 (0.95-1.07)	0.716	1.01 (0.95-1.07)	0.769
CP ≥1 (2 years)	8799/23,686 (37.2%)	33,375/94,744 (35.2%)	1.09 (1.06-1.12)	<0.001*	1.09 (1.06-1.13)	<0.001*	1.09 (1.06-1.12)	<0.001*
Men (n = 73,585)								
CP ≥1 (1 year)	3862/14,717 (26.2%)	14,229/58,868 (24.2%)	1.12 (1.07-1.16)	<0.001*	1.13 (1.08-1.17)	<0.001*	1.13 (1.08-1.17)	<0.001*
CP ≥2 (1 year)	1929/14,717 (13.1%)	7342/58,868 (12.5%)	1.06 (1.00-1.12)	0.038*	1.07 (1.01-1.13)	0.019*	1.07 (1.01-1.13)	0.018*
CP ≥3 (1 year)	1078/14,717 (7.3%)	4211/58,868 (7.2%)	1.03 (0.96-1.10)	0.468	1.03 (0.97-1.11)	0.334	1.04 (0.97-1.11)	0.328
CP ≥1 (2 years)	5891/14,717 (40.0%)	21,788/58,868 (37.0%)	1.00 (0.94-1.06)	0.967	1.00 (0.95-1.06)	0.893	1.00 (0.95-1.06)	0.885
Women (n = 143,210)								
CP ≥1 (1 year)	5924/28,642 (20.7%)	22,150/114,568 (19.3%)	1.09 (1.05-1.12)	<0.001*	1.09 (1.06-1.13)	<0.001*	1.09 (1.06-1.13)	<0.001*
CP ≥2 (1 year)	2787/28,642 (9.7%)	10,807/114,568 (9.4%)	1.04 (0.99-1.08)	0.121	1.04 (0.99-1.09)	0.084	1.04 (0.99-1.09)	0.083
CP ≥3 (1 year)	1506/28,642 (5.3%)	6031/114,568 (5.3%)	1.14 (1.09-1.18)	<0.001*	1.15 (1.11-1.19)	<0.001*	1.15 (1.10-1.19)	<0.001*
CP ≥1 (2 years)	9186/28,642 (32.1%)	35,062/114,568 (30.6%)	1.07 (1.04-1.10)	<0.001*	1.08 (1.05-1.11)	<0.001*	1.07 (1.05-1.11)	<0.001*
Low income (n = 105,870)								
CP ≥1 (1 year)	4500/21,174 (21.3%)	16,438/84,696 (19.4%)	1.12 (1.08-1.16)	<0.001*	1.13 (1.09-1.17)	<0.001*	1.13 (1.09-1.17)	<0.001*
CP ≥2 (1 year)	2154/21,174 (10.2%)	8008/84,696 (9.5%)	1.08 (1.03-1.14)	0.002*	1.09 (1.04-1.15)	0.001*	1.09 (1.04-1.15)	0.001*
CP ≥3 (1 year)	1187/21,174 (5.6%)	4504/84,696 (5.3%)	1.06 (0.99-1.13)	0.096	1.07 (1.00-1.14)	0.059	1.07 (1.00-1.14)	0.058
CP ≥1 (2 years)	7028/21,174 (33.2%)	26,018/84,696 (30.7%)	1.12 (1.09-1.16)	<0.001*	1.13 (1.09-1.17)	<0.001*	1.13 (1.09-1.16)	<0.001*
High income (n = 110,925)								
CP ≥1 (1 year)	5286/22,185 (23.8%)	19,941/88,740 (22.5%)	1.08 (1.04-1.12)	<0.001*	1.09 (1.05-1.12)	<0.001*	1.09 (1.05-1.12)	<0.001*
CP ≥2 (1 year)	2562/22,185 (11.6%)	10,141/88,740 (11.4%)	1.01 (0.97-1.06)	0.613	1.02 (0.97-1.07)	0.469	1.02 (0.97-1.07)	0.468
CP ≥3 (1 year)	1397/22,185 (6.3%)	5738/88,740 (6.5%)	0.97 (0.92-1.03)	0.362	0.98 (0.92-1.04)	0.473	0.98 (0.92-1.04)	0.477
CP ≥1 (2 years)	8049/22,185 (36.3%)	30,832/88,740 (34.7%)	1.07 (1.04-1.10)	<0.001*	1.08 (1.04-1.11)	<0.001*	1.08 (1.04-1.11)	<0.001*

Urban residents (n = 84,995)								
CP ≥1 (1 year)	4353/16,999 (25.6%)	15,839/67,996 (23.3%)	1.13 (1.09-1.18)	<0.001*	1.14 (1.10-1.19)	<0.001*	1.14 (1.10-1.18)	<0.001*
CP ≥2 (1 year)	2134/16,999 (12.6%)	8169/67,996 (12.0%)	1.05 (1.00-1.11)	0.054	1.06 (1.01-1.11)	0.029*	1.06 (1.00-1.11)	0.037*
CP ≥3 (1 year)	1181/16,999 (7.0%)	4695/67,996 (6.9%)	1.01 (0.94-1.08)	0.844	1.01 (0.95-1.08)	0.671	1.01 (0.95-1.08)	0.72
CP ≥1 (2 years)	6532/16,999 (38.4%)	24,195/67,996 (35.6%)	1.13 (1.09-1.17)	<0.001*	1.14 (1.10-1.18)	<0.001*	1.14 (1.10-1.18)	<0.001*
Rural residents (n = 131,800)								
CP ≥1 (1 year)	5433/26,360 (20.6%)	20,540/105,440 (19.5%)	1.09 (1.06-1.12)	<0.001*	1.09 (1.06-1.12)	<0.001*	1.09 (1.06-1.12)	<0.001*
CP ≥2 (1 year)	2582/26,360 (9.8%)	9980/105,440 (9.5%)	1.04 (0.99-1.09)	0.1	1.04 (1.00-1.09)	0.065	1.05 (1.00-1.09)	0.055
CP ≥3 (1 year)	1403/26,360 (5.3%)	5547/105,440 (5.3%)	1.01 (0.95-1.08)	0.688	1.02 (0.96-1.08)	0.546	1.02 (0.96-1.08)	0.509
CP ≥1 (2 years)	8545/26,360 (32.4%)	32,655/105,440 (31.0%)	1.07 (1.04-1.10)	<0.001*	1.07 (1.04-1.11)	<0.001*	1.08 (1.04-1.11)	<0.001*

CCI, Charlson Comorbidity Index; CP, chronic periodontitis; DBP, Diastolic blood pressure; SBP, Systolic blood pressure.

*Conditional or unconditional logistic regression analysis, significance at P <0.05.

†Stratified model for age, sex, income, and geographic region.

‡Model 1 was adjusted for smoking status, alcohol use, obesity, and CCI scores.

§Model 2 was adjusted for model 1 plus total cholesterol, SBP, DBP, and fasting blood glucose.
