# Supplementary material for: Association of Chronic Periodontitis with Migraine in a Korean Adult Population: A Nationwide Nested Case-Control Study
Source: Healthcare (Basel). 2025 Aug 26;13(17):2123. doi: 10.3390/healthcare13172123 (PMC12428593; doi:10.3390/healthcare13172123)
Supplement: Supplementary file 1 [file healthcare-13-02123-s001.zip › Table S2 (Migraine) - d.pdf]

Table S2. Subgroup analyses of crude and adjusted odds ratios according to obesity, smoking status, and alcohol consumption

Characteristics	No. of case (exposure/total, %)	No. of control (exposure/total, %)	Odds ratios for migraine (95% confidence interval) Crude[†]	P-value	Model 1[†‡]	P-value	Model 2[†§]	P-value
Underweight (n = 5457)								
CP ≥1 (1 year)	216/1046 (20.7%)	762/4411 (17.3%)	1.25 (1.05-1.48)	0.011*	1.26 (1.06-1.49)	0.009*	1.25 (1.05-1.48)	0.012*
CP ≥2 (1 year)	93/1046 (8.9%)	365/4411 (8.3%)	1.08 (0.85-1.37)	0.518	1.08 (0.85-1.38)	0.518	1.07 (0.84-1.36)	0.571
CP ≥3 (1 year)	48/1046 (4.6%)	198/4411 (4.5%)	1.02 (0.74-1.41)	0.888	1.02 (0.74-1.41)	0.898	1.02 (0.73-1.40)	0.93
CP ≥1 (2 years)	338/1046 (32.3%)	1197/4411 (27.1%)	1.28 (1.11-1.48)	0.001*	1.30 (1.12-1.50)	0.001*	1.29 (1.11-1.49)	0.001*
Normal weight (n = 77,867)								
CP ≥1 (1 year)	3377/15,442 (21.9%)	12,714/62,425 (20.4%)	1.09 (1.05-1.14)	<0.001*	1.09 (1.05-1.14)	<0.001*	1.09 (1.05-1.14)	<0.001*
CP ≥2 (1 year)	1615/15,442 (10.5%)	6259/62,425 (10.0%)	1.05 (0.99-1.11)	0.107	1.05 (0.99-1.11)	0.122	1.05 (0.99-1.11)	0.124
CP ≥3 (1 year)	892/15,442 (5.8%)	3503/62,425 (5.6%)	1.03 (0.96-1.11)	0.423	1.03 (0.96-1.11)	0.44	1.03 (0.95-1.11)	0.449
CP ≥1 (2 years)	5175/15,442 (33.5%)	20,030/62,425 (32.1%)	1.07 (1.03-1.11)	0.001*	1.07 (1.03-1.11)	0.001*	1.07 (1.03-1.11)	0.001*
Overweight (n = 58,023)								
CP ≥1 (1 year)	2714/11,743 (23.1%)	10,089/46,280 (21.8%)	1.08 (1.03-1.13)	0.002*	1.09 (1.04-1.14)	0.001*	1.09 (1.04-1.14)	0.001*
CP ≥2 (1 year)	1331/11,743 (11.3%)	5013/46,280 (10.8%)	1.05 (0.99-1.12)	0.119	1.06 (1.00-1.13)	0.069	1.06 (1.00-1.13)	0.071
CP ≥3 (1 year)	702/11,743 (6.0%)	2829/46,280 (6.1%)	0.98 (0.90-1.06)	0.588	0.99 (0.91-1.08)	0.764	0.99 (0.90-1.07)	0.731
CP ≥1 (2 years)	4197/11,743 (35.7%)	15,609/46,280 (33.7%)	1.09 (1.05-1.14)	<0.001*	1.10 (1.06-1.15)	<0.001*	1.10 (1.06-1.15)	<0.001*
Obese (n = 75,448Z								
CP ≥1 (1 year)	3479/15,128 (23.0%)	12,814/60,320 (21.2%)	1.11 (1.06-1.16)	<0.001*	1.12 (1.07-1.17)	<0.001*	1.12 (1.07-1.17)	<0.001*
CP ≥2 (1 year)	1677/15,128 (11.1%)	6512/60,320 (10.8%)	1.03 (0.97-1.09)	0.302	1.04 (0.99-1.11)	0.147	1.04 (0.99-1.11)	0.139
CP ≥3 (1 year)	942/15,128 (6.2%)	3712/60,320 (6.2%)	1.01 (0.94-1.09)	0.738	1.03 (0.95-1.11)	0.5	1.03 (0.96-1.11)	0.46
CP ≥1 (2 years)	5367/15,128 (35.5%)	20,014/60,320 (33.2%)	1.11 (1.07-1.15)	<0.001*	1.12 (1.08-1.17)	<0.001*	1.12 (1.08-1.16)	<0.001*
Non-smoker (n = 170,834)								
CP ≥1 (1 year)	7456/34,538 (21.6%)	27,509/136,296 (20.2%)	1.09 (1.06-1.12)	<0.001*	1.09 (1.06-1.12)	<0.001*	1.09 (1.06-1.12)	<0.001*
CP ≥2 (1 year)	3553/34,538 (10.3%)	13,556/136,296 (10.0%)	1.04 (1.00-1.08)	0.057	1.04 (1.00-1.08)	0.044*	1.04 (1.00-1.08)	0.045*
CP ≥3 (1 year)	1930/34,538 (5.6%)	7573/136,296 (5.6%)	1.01 (0.96-1.06)	0.818	1.01 (0.96-1.06)	0.737	1.01 (0.96-1.06)	0.73
CP ≥1 (2 years)	11,474/34,538 (33.2%)	43,104/136,296 (31.6%)	1.08 (1.05-1.10)	<0.001*	1.08 (1.05-1.11)	<0.001*	1.08 (1.05-1.11)	<0.001*
Past and current smoker (n = 45,961)								
CP ≥1 (1 year)	2330/8,821 (26.4%)	8,870/37,140 (23.9%)	1.14 (1.08-1.21)	<0.001*	1.15 (1.09-1.21)	<0.001*	1.15 (1.09-1.21)	<0.001*
CP ≥2 (1 year)	1,163/8,821 (13.2%)	4,593/37,140 (12.4%)	1.08 (1.00-1.15)	0.037*	1.08 (1.01-1.16)	0.030*	1.08 (1.01-1.16)	0.027*
CP ≥3 (1 year)	654/8,821 (7.4%)	2,669/37,140 (7.2%)	1.03 (0.95-1.13)	0.454	1.04 (0.95-1.14)	0.376	1.04 (0.95-1.14)	0.374
CP ≥1 (2 years)	3,603/8,821 (40.9%)	13,746/37,140 (37.0%)	1.18 (1.12-1.23)	<0.001*	1.18 (1.13-1.24)	<0.001*	1.18 (1.13-1.24)	<0.001*
Alcohol consumption <1 time a week (n = 174,107)								

CP ≥1 (1 year)	7741/35,332 (21.9%)	28,327/138,775 (20.4%)	1.09 (1.06-1.13)	<0.001*	1.10 (1.07-1.13)	<0.001*	1.10 (1.07-1.13)	<0.001*
CP ≥2 (1 year)	3702/35,332 (10.5%)	14,091/138,775 (10.2%)	1.04 (1.00-1.08)	0.07	1.04 (1.00-1.08)	0.06	1.04 (1.00-1.08)	0.055
CP ≥3 (1 year)	2011/35,332 (5.7%)	7889/138,775 (5.7%)	1.00 (0.95-1.05)	0.96	1.00 (0.95-1.06)	0.895	1.00 (0.96-1.06)	0.859
CP ≥1 (2 years)	11,947/35,332 (33.8%)	44,478/138,775 (32.1%)	1.08 (1.06-1.11)	<0.001*	1.09 (1.06-1.11)	<0.001*	1.09 (1.06-1.11)	<0.001*
Alcohol consumption ≥1 time a week (n = 42,688)								
CP ≥1 (1 year)	2045/8027 (25.5%)	8052/34,661 (23.2%)	1.13 (1.07-1.19)	<0.001*	1.14 (1.08-1.21)	<0.001*	1.14 (1.07-1.20)	<0.001*
CP ≥2 (1 year)	1014/8027 (12.6%)	4058/34,661 (11.7%)	1.09 (1.01-1.17)	0.021*	1.10 (1.02-1.18)	0.012*	1.10 (1.02-1.18)	0.013*
CP ≥3 (1 year)	573/8,027 (7.1%)	2353/34,661 (6.8%)	1.06 (0.96-1.16)	0.264	1.07 (0.97-1.17)	0.188	1.06 (0.97-1.17)	0.207
CP ≥1 (2 years)	3,130/8,027 (39.0%)	12,372/34,661 (35.7%)	1.15 (1.10-1.21)	<0.001*	1.16 (1.11-1.22)	<0.001*	1.16 (1.10-1.22)	<0.001*

CCI, Charlson Comorbidity Index; CP, chronic periodontitis; DBP, Diastolic blood pressure; SBP, Systolic blood pressure.

*Conditional or unconditional logistic regression analysis, significance at P <0.05.

†Stratified model for age, sex, income, and geographic region.

‡Model 1 was adjusted for smoking status, alcohol use, obesity, and CCI scores.

§Model 2 was adjusted for model 1 plus total cholesterol, SBP, DBP, and fasting blood glucose.
