# Supplementary material for: Association of Chronic Periodontitis with Migraine in a Korean Adult Population: A Nationwide Nested Case-Control Study
Source: Healthcare (Basel). 2025 Aug 26;13(17):2123. doi: 10.3390/healthcare13172123 (PMC12428593; doi:10.3390/healthcare13172123)
Supplement: Supplementary file 1 [file healthcare-13-02123-s001.zip › Table S3 (Migraine) - d.pdf]

Table S3. Subgroup analyses of crude and adjusted odds ratios according to blood pressure, fasting blood glucose, total cholesterol, and CCI scores

Characteristics	No. of case (exposure/total, %)	No. of control (exposure/total, %)	Odds ratios for migraine (95% confidence interval)					
			Crude†	P-value	Model 1†‡	P-value	Model 2†§	P-value
SBP <140 mmHg and DBP <90 mmHg (n = 61,633)								
CP ≥1 (1 year)	2944/12,692 (23.2%)	10,749/48,941 (22.0%)	1.07 (1.02-1.12)	0.003*	1.07 (1.02-1.12)	0.005*	1.07 (1.02-1.12)	0.004*
CP ≥2 (1 year)	1407/12,692 (11.1%)	5392/48,941 (11.0%)	1.01 (0.95-1.07)	0.826	1.00 (0.94-1.07)	0.955	1.01 (0.95-1.07)	0.83
CP ≥3 (1 year)	777/12,692 (6.1%)	3093/48,941 (6.3%)	0.97 (0.89-1.05)	0.417	0.96 (0.89-1.04)	0.356	0.97 (0.89-1.05)	0.439
CP ≥1 (2 years)	4526/12,692 (35.7%)	16,661/48,941 (34.0%)	1.07 (1.03-1.12)	0.001*	1.07 (1.03-1.11)	0.001*	1.07 (1.03-1.12)	0.001*
SBP ≥140 mmHg or DBP ≥90 mmHg (n = 155,162)								
CP ≥1 (1 year)	6842/30,667 (22.3%)	25,630/124,495 (20.6%)	1.11 (1.07-1.14)	<0.001*	1.12 (1.08-1.15)	<0.001*	1.12 (1.08-1.15)	<0.001*
CP ≥2 (1 year)	3309/30,667 (10.8%)	12,757/124,495 (10.3%)	1.06 (1.02-1.10)	0.005*	1.07 (1.03-1.11)	0.001*	1.07 (1.03-1.11)	0.001*
CP ≥3 (1 year)	1807/30,667 (5.9%)	7149/124,495 (5.7%)	1.03 (0.97-1.08)	0.31	1.04 (0.98-1.10)	0.165	1.04 (0.98-1.10)	0.165
CP ≥1 (2 years)	10,551/30,667 (34.4%)	40,189/124,495 (32.3%)	1.10 (1.07-1.13)	<0.001*	1.11 (1.08-1.14)	<0.001*	1.11 (1.08-1.14)	<0.001*
Fasting blood glucose <100 mg/dL (n = 138,318)								
CP ≥1 (1 year)	6251/28,602 (21.9%)	21,957/109,716 (20.0%)	1.12 (1.08-1.15)	<0.001*	1.12 (1.09-1.16)	<0.001*	1.12 (1.08-1.15)	<0.001*
CP ≥2 (1 year)	2976/28,602 (10.4%)	10,711/109,716 (9.8%)	1.07 (1.03-1.12)	0.001*	1.08 (1.03-1.12)	0.001*	1.07 (1.03-1.12)	0.002*
CP ≥3 (1 year)	1588/28,602 (5.6%)	6009/109,716 (5.5%)	1.01 (0.96-1.07)	0.618	1.02 (0.96-1.08)	0.547	1.01 (0.96-1.07)	0.674
CP ≥1 (2 years)	9608/28,602 (33.6%)	34,582/109,716 (31.5%)	1.10 (1.07-1.13)	<0.001*	1.10 (1.07-1.13)	<0.001*	1.10 (1.07-1.13)	<0.001*
Fasting blood glucose ≥100 mg/dL (n = 78,477)								
CP ≥1 (1 year)	3535/14,757 (24.0%)	14,422/63,720 (22.6%)	1.08 (1.03-1.12)	0.001*	1.09 (1.04-1.13)	<0.001*	1.08 (1.04-1.13)	<0.001*
CP ≥2 (1 year)	1,740/14,757 (11.8%)	7438/63,720 (11.7%)	1.01 (0.96-1.07)	0.687	1.02 (0.96-1.08)	0.479	1.01 (0.96-1.07)	0.626
CP ≥3 (1 year)	996/14,757 (6.8%)	4233/63,720 (6.6%)	1.02 (0.95-1.09)	0.64	1.03 (0.96-1.10)	0.465	1.02 (0.95-1.10)	0.566
CP ≥1 (2 years)	5469/14,757 (37.1%)	22,268/63,720 (35.0%)	1.10 (1.06-1.14)	<0.001*	1.11 (1.07-1.15)	<0.001*	1.10 (1.06-1.14)	<0.001*
Total cholesterol <200mg/dL (n = 113,523)								
CP ≥1 (1 year)	5232/22,632 (23.1%)	19,351/90,891 (21.3%)	1.11 (1.07-1.15)	<0.001*	1.12 (1.08-1.16)	<0.001*	1.11 (1.08-1.15)	<0.001*
CP ≥2 (1 year)	2510/22,632 (11.1%)	9753/90,891 (10.7%)	1.04 (0.99-1.09)	0.115	1.04 (0.99-1.09)	0.092	1.04 (0.99-1.09)	0.097
CP ≥3 (1 year)	1374/22,632 (6.1%)	5502/90,891 (6.1%)	1.00 (0.94-1.07)	0.921	1.01 (0.95-1.07)	0.815	1.01 (0.95-1.07)	0.844
CP ≥1 (2 years)	8032/22,632 (35.5%)	30,065/90,891 (33.1%)	1.11 (1.08-1.15)	<0.001*	1.12 (1.09-1.15)	<0.001*	1.12 (1.08-1.15)	<0.001*
Total cholesterol ≥200mg/dL (n = 103,272)								
CP ≥1 (1 year)	4554/20,727 (22.0%)	17,028/82,545 (20.6%)	1.08 (1.04-1.12)	<0.001*	1.09 (1.05-1.13)	<0.001*	1.09 (1.05-1.13)	<0.001*
CP ≥2 (1 year)	2206/20,727 (10.6%)	8396/82,545 (10.2%)	1.05 (1.00-1.11)	0.046*	1.06 (1.01-1.11)	0.020*	1.06 (1.01-1.12)	0.020*
CP ≥3 (1 year)	1210/20,727 (5.8%)	4740/82,545 (5.7%)	1.02 (0.95-1.09)	0.596	1.03 (0.96-1.10)	0.423	1.03 (0.96-1.10)	0.408

CP ≥1 (2 years)	7045/20,727 (34.0%)	26,785/82,545 (32.5%)	1.07 (1.04-1.11)	<0.001*	1.08 (1.05-1.12)	<0.001*	1.08 (1.04-1.11)	<0.001*
CCI scores = 0 (n = 129,881)								
CP ≥1 (1 year)	5535/23,948 (23.1%)	22,945/105,933 (21.7%)	1.09 (1.05-1.12)	<0.001*	1.09 (1.05-1.12)	<0.001*	1.09 (1.05-1.12)	<0.001*
CP ≥2 (1 year)	2706/23,948 (11.3%)	11,564/105,933 (10.9%)	1.04 (1.00-1.09)	0.084	1.04 (0.99-1.08)	0.11	1.04 (0.99-1.09)	0.11
CP ≥3 (1 year)	1502/23,948 (6.3%)	6606/105,933 (6.2%)	1.01 (0.95-1.07)	0.835	1.00 (0.95-1.06)	0.883	1.00 (0.95-1.07)	0.882
CP ≥1 (2 years)	8489/23,948 (35.5%)	35,509/105,933 (33.5%)	1.09 (1.06-1.12)	<0.001*	1.09 (1.06-1.12)	<0.001*	1.09 (1.06-1.12)	<0.001*
CCI score = 1 (n = 36,147)								
CP ≥1 (1 year)	1909/8,581 (22.3%)	5646/27,566 (20.5%)	1.11 (1.05-1.18)	<0.001*	1.13 (1.06-1.20)	<0.001*	1.13 (1.07-1.20)	<0.001*
CP ≥2 (1 year)	899/8,581 (10.5%)	2803/27,566 (10.2%)	1.03 (0.96-1.12)	0.408	1.05 (0.97-1.14)	0.221	1.06 (0.98-1.15)	0.163
CP ≥3 (1 year)	479/8,581 (5.6%)	1553/27,566 (5.6%)	0.99 (0.89-1.10)	0.856	1.01 (0.91-1.12)	0.901	1.02 (0.91-1.13)	0.758
CP ≥1 (2 years)	2944/8,581 (34.3%)	9000/27,566 (32.7%)	1.08 (1.02-1.13)	0.004*	1.10 (1.04-1.16)	<0.001*	1.10 (1.05-1.16)	<0.001*
CCI score ≥2 (n = 50,767)								
CP ≥1 (1 year)	2342/10,830 (21.6%)	7788/39,937 (19.5%)	1.14 (1.08-1.20)	<0.001*	1.14 (1.08-1.20)	<0.001*	1.14 (1.08-1.20)	<0.001*
CP ≥2 (1 year)	1111/10,830 (10.3%)	3782/39,937 (9.5%)	1.09 (1.02-1.17)	0.014*	1.09 (1.02-1.17)	0.013*	1.09 (1.02-1.17)	0.014*
CP ≥3 (1 year)	603/10,830 (5.6%)	2083/39,937 (5.2%)	1.07 (0.98-1.18)	0.147	1.07 (0.98-1.18)	0.15	1.07 (0.97-1.18)	0.159
CP ≥1 (2 years)	3644/10,830 (33.7%)	12,341/39,937 (30.9%)	1.13 (1.08-1.19)	<0.001*	1.13 (1.08-1.19)	<0.001*	1.13 (1.08-1.19)	<0.001*

CCI, Charlson Comorbidity Index; CP, chronic periodontitis; DBP, Diastolic blood pressure; SBP, Systolic blood pressure.

*Conditional or unconditional logistic regression analysis, significance at P <0.05.

†Stratified model for age, sex, income, and geographic region.

‡Model 1 was adjusted for smoking status, alcohol use, obesity, and CCI scores.

§Model 2 was adjusted for model 1 plus total cholesterol, SBP, DBP, and fasting blood glucose.
