# Supplementary material for: Association of Chronic Periodontitis with Migraine in a Korean Adult Population: A Nationwide Nested Case-Control Study
Source: Healthcare (Basel). 2025 Aug 26;13(17):2123. doi: 10.3390/healthcare13172123 (PMC12428593; doi:10.3390/healthcare13172123)
Supplement: Supplementary file 1 [file healthcare-13-02123-s001.zip › Table S4 (Migraine with aura) - d.pdf]

Table S4. Subgroup analyses of crude and adjusted odds ratios according to age, sex, income and region of residence

Characteristics	No. of case (exposure/total, %)	No. of control (exposure/total, %)	Odds ratios for migraine with aura (95% confidence interval)					
			Crude†	P-value	Model 1†‡	P-value	Model 2†§	P-value
Age < 60 years old (n = 8465)								
CP ≥1 (1 year)	341/1693 (20.1%)	1198/6772 (17.7%)	1.17 (1.03-1.34)	0.019*	1.19 (1.03-1.36)	0.014*	1.18 (1.03-1.35)	0.016*
CP ≥2 (1 year)	154/1693 (9.1%)	575/6772 (8.5%)	1.08 (0.90-1.30)	0.427	1.09 (0.90-1.31)	0.378	1.09 (0.90-1.31)	0.381
CP ≥3 (1 year)	78/1693 (4.6%)	309/6772 (4.6%)	1.01 (0.78-1.30)	0.938	1.02 (0.79-1.32)	0.876	1.02 (0.79-1.32)	0.885
CP ≥1 (2 years)	542/1693 (32.0%)	1900/6772 (28.1%)	1.21 (1.08-1.35)	0.001*	1.22 (1.09-1.38)	0.001*	1.22 (1.09-1.37)	0.001*
Age ≥ 60 years old (n = 7295)								
CP ≥1 (1 year)	347/1459 (23.8%)	1263/5836 (21.6%)	1.13 (0.99-1.29)	0.078	1.15 (1.00-1.32)	0.049*	1.14 (1.00-1.31)	0.059
CP ≥2 (1 year)	160/1459 (11.0%)	641/5836 (11.0%)	1.00 (0.83-1.20)	0.985	1.02 (0.84-1.22)	0.869	1.01 (0.84-1.21)	0.942
CP ≥3 (1 year)	73/1459 (5.0%)	350/5836 (6.0%)	0.83 (0.64-1.07)	0.147	0.83 (0.64-1.08)	0.162	0.82 (0.63-1.06)	0.13
CP ≥1 (2 years)	525/1459 (36.0%)	1936/5,836 (33.2%)	1.13 (1.00-1.28)	0.042*	1.16 (1.02-1.31)	0.019*	1.15 (1.01-1.29)	0.029*
Men (n = 4805)								
CP ≥1 (1 year)	262/961 (27.3%)	867/3844 (22.6%)	1.29 (1.10-1.51)	0.002*	1.31 (1.12-1.54)	0.001*	1.31 (1.11-1.54)	0.001*
CP ≥2 (1 year)	136/961 (14.2%)	436/3844 (11.3%)	1.29 (1.05-1.58)	0.016*	1.32 (1.07-1.63)	0.009*	1.31 (1.07-1.62)	0.011*
CP ≥3 (1 year)	69/961 (7.2%)	250/3844 (6.5%)	1.11 (0.84-1.47)	0.451	1.13 (0.86-1.50)	0.375	1.12 (0.85-1.48)	0.437
CP ≥1 (2 years)	401/961 (41.7%)	1355/3844 (35.3%)	1.32 (1.14-1.52)	0.000*	1.34 (1.16-1.55)	<0.001*	1.34 (1.16-1.55)	<0.001*
Women (n = 10,955)								
CP ≥1 (1 year)	426/2191 (19.4%)	1594/8764 (18.2%)	1.09 (0.96-1.22)	0.176	1.10 (0.97-1.23)	0.136	1.10 (0.97-1.24)	0.132
CP ≥2 (1 year)	178/2191 (8.1%)	780/8764 (8.9%)	0.91 (0.76-1.07)	0.25	0.91 (0.77-1.08)	0.293	0.92 (0.77-1.09)	0.31
CP ≥3 (1 year)	82/2191 (3.7%)	409/8764 (4.7%)	0.79 (0.62-1.01)	0.062	0.80 (0.63-1.02)	0.068	0.80 (0.63-1.02)	0.068
CP ≥1 (2 years)	666/2191 (30.4%)	2481/8764 (28.3%)	1.11 (1.00-1.22)	0.053	1.12 (1.01-1.24)	0.033*	1.12 (1.01-1.24)	0.037*
Low income (n = 7700)								
CP ≥1 (1 year)	289/1540 (18.8%)	1142/6160 (18.5%)	1.02 (0.88-1.17)	0.837	1.02 (0.89-1.18)	0.746	1.03 (0.89-1.19)	0.724
CP ≥2 (1 year)	128/1540 (8.3%)	534/6160 (8.7%)	0.96 (0.78-1.17)	0.658	0.96 (0.79-1.18)	0.727	0.97 (0.79-1.18)	0.738
CP ≥3 (1 year)	63/1540 (4.1%)	287/6160 (4.7%)	0.87 (0.66-1.15)	0.339	0.88 (0.66-1.16)	0.362	0.88 (0.67-1.17)	0.38
CP ≥1 (2 years)	483/1540 (31.4%)	1768/6160 (28.7%)	1.14 (1.01-1.28)	0.040*	1.15 (1.02-1.30)	0.025*	1.15 (1.02-1.30)	0.026*
High income (n = 8060)								
CP ≥1 (1 year)	399/1612 (24.8%)	1319/6448 (20.5%)	1.28 (1.13-1.45)	0.000*	1.30 (1.14-1.48)	<0.001*	1.29 (1.14-1.47)	<0.001*
CP ≥2 (1 year)	186/1612 (11.5%)	682/6448 (10.6%)	1.10 (0.93-1.31)	0.266	1.12 (0.94-1.33)	0.202	1.12 (0.94-1.33)	0.219
CP ≥3 (1 year)	88/1612 (5.5%)	372/6448 (5.8%)	0.94 (0.74-1.20)	0.631	0.96 (0.75-1.22)	0.717	0.95 (0.74-1.20)	0.658
CP ≥1 (2 years)	584/1,612 (36.2%)	2068/6448 (32.1%)	1.20 (1.07-1.35)	0.002*	1.23 (1.09-1.37)	0.001*	1.22 (1.09-1.37)	0.001*
Urban residents (n = 6165)								

CP ≥1 (1 year)	289/1233 (23.4%)	1102/4932 (22.3%)	1.06 (0.92-1.23)	0.411	1.08 (0.93-1.25)	0.33	1.07 (0.92-1.24)	0.381
CP ≥2 (1 year)	144/1233 (11.7%)	580/4932 (11.8%)	0.99 (0.82-1.20)	0.937	1.01 (0.83-1.23)	0.935	1.00 (0.82-1.22)	0.972
CP ≥3 (1 year)	69/1233 (5.6%)	331/4932 (6.7%)	0.82 (0.63-1.08)	0.156	0.83 (0.63-1.08)	0.164	0.82 (0.63-1.08)	0.153
CP ≥1 (2 years)	451/1233 (36.6%)	1653/4932 (33.5%)	1.14 (1.00-1.30)	0.043*	1.17 (1.02-1.33)	0.023*	1.16 (1.02-1.32)	0.028*
			Rural residents (n = 9595)					
CP ≥1 (1 year)	399/1919 (20.8%)	1359/7676 (17.7%)	1.22 (1.08-1.38)	0.002*	1.24 (1.09-1.40)	0.001*	1.24 (1.09-1.41)	0.001*
CP ≥2 (1 year)	170/1919 (8.9%)	636/7676 (8.3%)	1.08 (0.90-1.28)	0.418	1.09 (0.91-1.30)	0.344	1.09 (0.91-1.30)	0.34
CP ≥3 (1 year)	82/1919 (4.3%)	328/7676 (4.3%)	1.00 (0.78-1.28)	1	1.02 (0.79-1.31)	0.885	1.01 (0.79-1.30)	0.927
CP ≥1 (2 years)	616/1919 (32.1%)	2183/7676 (28.4%)	1.19 (1.07-1.33)	0.002*	1.21 (1.08-1.35)	0.001*	1.21 (1.08-1.35)	0.001*

CCI, Charlson Comorbidity Index; CP, chronic periodontitis; DBP, Diastolic blood pressure; SBP, Systolic blood pressure.

*Conditional or unconditional logistic regression analysis, significance at P <0.05.

†Stratified model for age, sex, income, and geographic region.

‡Model 1 was adjusted for smoking status, alcohol use, obesity, and CCI scores.

§Model 2 was adjusted for model 1 plus total cholesterol, SBP, DBP, and fasting blood glucose.
