# Supplementary material for: Association of Chronic Periodontitis with Migraine in a Korean Adult Population: A Nationwide Nested Case-Control Study
Source: Healthcare (Basel). 2025 Aug 26;13(17):2123. doi: 10.3390/healthcare13172123 (PMC12428593; doi:10.3390/healthcare13172123)
Supplement: Supplementary file 1 [file healthcare-13-02123-s001.zip › Table S5 (Migraine with aura) - d.pdf]

Table S5. Subgroup analyses of crude and adjusted odds ratios according to obesity, smoking status, and alcohol consumption

Characteristics	No. of case	No. of control	Odds ratios for migraine with aura (95% confidence interval)					
	(exposure/total, %)	(exposure/total, %)	Crude[†]	P-value	Model 1[†‡]	P-value	Model 2[†§]	P-value
Underweight (n = 380)								
CP ≥1 (1 year)	17/88 (19.3%)	51/292 (17.5%)	1.13 (0.62-2.08)	0.691	1.15 (0.62-2.14)	0.666	1.16 (0.62-2.18)	0.647
CP ≥2 (1 year)	6/88 (6.8%)	22/292 (7.5%)	0.90 (0.35-2.29)	0.822	0.83 (0.32-2.15)	0.702	0.85 (0.32-2.21)	0.731
CP ≥3 (1 year)	5/88 (5.7%)	14/292 (4.8%)	1.20 (0.42-3.42)	0.738	1.07 (0.37-3.14)	0.898	1.09 (0.37-3.22)	0.877
CP ≥1 (2 years)	23/88 (26.1%)	72/292 (24.7%)	1.08 (0.63-1.86)	0.779	1.11 (0.63-1.94)	0.717	1.10 (0.63-1.94)	0.739
Normal weight (n = 5676)								
CP ≥1 (1 year)	244/1167 (20.9%)	846/4509 (18.8%)	1.14 (0.98-1.34)	0.097	1.15 (0.98-1.35)	0.084	1.14 (0.97-1.34)	0.109
CP ≥2 (1 year)	102/1167 (8.7%)	421/4509 (9.3%)	0.93 (0.74-1.17)	0.53	0.93 (0.74-1.16)	0.512	0.91 (0.73-1.15)	0.44
CP ≥3 (1 year)	52/1167 (4.5%)	234/4509 (5.2%)	0.85 (0.63-1.16)	0.308	0.84 (0.62-1.15)	0.28	0.83 (0.61-1.13)	0.236
CP ≥1 (2 years)	366/1167 (31.4%)	1337/4509 (29.7%)	1.08 (0.94-1.25)	0.256	1.09 (0.95-1.26)	0.221	1.08 (0.94-1.25)	0.268
Overweight (n = 4260)								
CP ≥1 (1 year)	204/864 (23.6%)	682/3396 (20.1%)	1.23 (1.03-1.47)	0.023*	1.26 (1.05-1.51)	0.012*	1.25 (1.05-1.50)	0.014*
CP ≥2 (1 year)	98/864 (11.3%)	320/3396 (9.4%)	1.23 (0.97-1.56)	0.091	1.27 (1.00-1.62)	0.054	1.28 (1.00-1.63)	0.048*
CP ≥3 (1 year)	46/864 (5.3%)	174/3396 (5.1%)	1.04 (0.75-1.45)	0.81	1.08 (0.77-1.51)	0.652	1.08 (0.77-1.50)	0.675
CP ≥1 (2 years)	304/864 (35.2%)	1039/3396 (30.6%)	1.23 (1.05-1.44)	0.010*	1.26 (1.07-1.47)	0.005*	1.25 (1.07-1.47)	0.006*
Obese (n = 5444)								
CP ≥1 (1 year)	223/1033 (21.6%)	882/4411 (20.0%)	1.10 (0.93-1.30)	0.252	1.12 (0.95-1.32)	0.193	1.13 (0.95-1.33)	0.158
CP ≥2 (1 year)	108/1033 (10.5%)	453/4411 (10.3%)	1.02 (0.82-1.27)	0.859	1.04 (0.83-1.29)	0.762	1.04 (0.83-1.30)	0.728
CP ≥3 (1 year)	48/1033 (4.7%)	237/4411 (5.4%)	0.86 (0.62-1.18)	0.346	0.87 (0.63-1.20)	0.387	0.87 (0.63-1.19)	0.383
CP ≥1 (2 years)	374/1033 (36.2%)	1388/4411 (31.5%)	1.24 (1.07-1.42)	0.003*	1.26 (1.09-1.45)	0.002*	1.27 (1.10-1.46)	0.001*
Non-smoker (n = 12,682)								
CP ≥1 (1 year)	525/2579 (20.4%)	1,908/10,103 (18.9%)	1.10 (0.99-1.22)	0.09	1.10 (0.99-1.23)	0.082	1.10 (0.99-1.22)	0.091
CP ≥2 (1 year)	229/2579 (8.9%)	938/10,103 (9.3%)	0.95 (0.82-1.11)	0.53	0.95 (0.82-1.11)	0.532	0.95 (0.82-1.11)	0.529
CP ≥3 (1 year)	108/2579 (4.2%)	499/10,103 (4.9%)	0.84 (0.68-1.04)	0.111	0.84 (0.68-1.04)	0.105	0.84 (0.68-1.04)	0.101
CP ≥1 (2 years)	819/2579 (31.8%)	2,957/10,103 (29.3%)	1.12 (1.02-1.23)	0.014*	1.13 (1.03-1.24)	0.011*	1.12 (1.02-1.24)	0.015*
Past and current smoker (n = 3078)								
CP ≥1 (1 year)	163/573 (28.5%)	553/2505 (22.1%)	1.40 (1.14-1.72)	0.001*	1.43 (1.17-1.76)	0.001*	1.44 (1.17-1.78)	0.001*
CP ≥2 (1 year)	85/573 (14.8%)	278/2505 (11.1%)	1.40 (1.07-1.81)	0.013*	1.43 (1.10-1.87)	0.008*	1.42 (1.09-1.85)	0.010*
CP ≥3 (1 year)	43/573 (7.5%)	160/2505 (6.4%)	1.19 (0.84-1.69)	0.332	1.21 (0.85-1.73)	0.278	1.17 (0.82-1.66)	0.39
CP ≥1 (2 years)	248/573 (43.3%)	879/2505 (35.1%)	1.41 (1.17-1.70)	<0.001*	1.45 (1.20-1.74)	<0.001*	1.46 (1.21-1.76)	<0.001*
Alcohol consumption <1 time a week (n = 12,837)								

CP ≥1 (1 year)	552/2633 (21.0%)	1950/10,204 (19.1%)	1.12 (1.01-1.25)	0.032*	1.13 (1.02-1.26)	0.022*	1.13 (1.02-1.26)	0.023*
CP ≥2 (1 year)	243/2633 (9.2%)	959/10,204 (9.4%)	0.98 (0.85-1.14)	0.791	0.99 (0.85-1.15)	0.871	0.99 (0.85-1.15)	0.88
CP ≥3 (1 year)	117/2633 (4.4%)	508/10,204 (5.0%)	0.89 (0.72-1.09)	0.256	0.89 (0.72-1.09)	0.268	0.89 (0.72-1.09)	0.256
CP ≥1 (2 years)	845/2633 (32.1%)	3,034/10,204 (29.7%)	1.12 (1.02-1.22)	0.019*	1.13 (1.03-1.24)	0.012*	1.12 (1.02-1.23)	0.014*
Alcohol consumption ≥1 time a week (n = 2923)								
CP ≥1 (1 year)	136/519 (26.2%)	511/2404(21.3%)	1.32 (1.06-1.64)	0.014*	1.33 (1.07-1.66)	0.011*	1.32 (1.06-1.65)	0.015*
CP ≥2 (1 year)	71/519 (13.7%)	257/2404(10.7%)	1.32 (1.00-1.76)	0.051	1.34 (1.01-1.79)	0.042*	1.33 (1.00-1.77)	0.05
CP ≥3 (1 year)	34/519 (6.6%)	151/2404 (6.3%)	1.05 (0.71-1.54)	0.817	1.05 (0.72-1.55)	0.787	1.04 (0.70-1.53)	0.853
CP ≥1 (2 years)	222/519 (42.8%)	802/2404 (33.4%)	1.49 (1.23-1.81)	<0.001*	1.52 (1.25-1.85)	<0.001*	1.52 (1.25-1.86)	<0.001*

CCI, Charlson Comorbidity Index; CP, chronic periodontitis; DBP, Diastolic blood pressure; SBP, Systolic blood pressure.

*Conditional or unconditional logistic regression analysis, significance at $P < 0.05$.

†Stratified model for age, sex, income, and geographic region.

‡Model 1 was adjusted for smoking status, alcohol use, obesity, and CCI scores.

§Model 2 was adjusted for model 1 plus total cholesterol, SBP, DBP, and fasting blood glucose.
