# Supplementary material for: Association of Chronic Periodontitis with Migraine in a Korean Adult Population: A Nationwide Nested Case-Control Study
Source: Healthcare (Basel). 2025 Aug 26;13(17):2123. doi: 10.3390/healthcare13172123 (PMC12428593; doi:10.3390/healthcare13172123)
Supplement: Supplementary file 1 [file healthcare-13-02123-s001.zip › Table S6 (Migraine with aura) - d.pdf]

Table S6. Subgroup analyses of crude and adjusted odds ratios according to blood pressure, fasting blood glucose, total cholesterol, and CCI scores

Characteristics	No. of case	No. of control	Odds ratios for migraine with aura (95% confidence interval)					
	(exposure/total, %)	(exposure/total, %)	Crude†	P-value	Model 1†‡	P-value	Model 2†§	P-value
SBP < 140 mmHg and DBP < 90 mmHg (n = 4785)								
CP ≥1 (1 year)	229/1032 (22.2%)	766/3753 (20.4%)	1.11 (0.94-1.31)	0.212	1.10 (0.93-1.30)	0.28	1.09 (0.92-1.29)	0.305
CP ≥2 (1 year)	104/1032 (10.1%)	383/3753 (10.2%)	0.99 (0.78-1.24)	0.905	0.97 (0.77-1.22)	0.774	0.97 (0.77-1.22)	0.77
CP ≥3 (1 year)	48/1032 (4.7%)	219/3753 (5.8%)	0.79 (0.57-1.08)	0.143	0.77 (0.55-1.06)	0.104	0.76 (0.55-1.05)	0.101
CP ≥1 (2 years)	358/1032 (34.7%)	1186/3753 (31.6%)	1.15 (0.99-1.33)	0.06	1.13 (0.98-1.31)	0.1	1.13 (0.97-1.30)	0.115
SBP ≥ 140 mmHg or DBP ≥ 90 mmHg (n = 10,975)								
CP ≥1 (1 year)	459/2120 (21.7%)	1695/8855 (19.1%)	1.17 (1.04-1.31)	0.009*	1.20 (1.06-1.35)	0.003*	1.20 (1.07-1.35)	0.002*
CP ≥2 (1 year)	210/2120 (9.9%)	833/8855 (9.4%)	1.06 (0.90-1.24)	0.482	1.09 (0.93-1.28)	0.292	1.10 (0.93-1.29)	0.262
CP ≥3 (1 year)	103/2120 (4.9%)	440/8855 (5.0%)	0.98 (0.78-1.22)	0.834	1.01 (0.81-1.26)	0.956	1.01 (0.81-1.25)	0.962
CP ≥1 (2 years)	709/2120 (33.4%)	2650/8855 (29.9%)	1.18 (1.06-1.30)	0.002*	1.21 (1.09-1.34)	<0.001*	1.21 (1.10-1.35)	<0.001*
Fasting blood glucose < 100 mg/dL (n = 10,451)								
CP ≥1 (1 year)	471/2195 (21.5%)	1,552/8256 (18.8%)	1.18 (1.05-1.33)	0.005*	1.19 (1.06-1.34)	0.004*	1.18 (1.05-1.33)	0.006*
CP ≥2 (1 year)	215/2195 (9.8%)	751/8256 (9.1%)	1.09 (0.93-1.27)	0.315	1.09 (0.93-1.28)	0.273	1.08 (0.92-1.27)	0.323
CP ≥3 (1 year)	103/2195 (4.7%)	417/8256 (5.1%)	0.93 (0.74-1.15)	0.493	0.93 (0.75-1.16)	0.526	0.92 (0.74-1.15)	0.45
CP ≥1 (2 years)	721/2195 (32.9%)	2452/8256 (29.7%)	1.16 (1.05-1.28)	0.004*	1.17 (1.06-1.29)	0.003*	1.16 (1.05-1.28)	0.005*
Fasting blood glucose ≥100 mg/dL (n = 5309)								
CP ≥1 (1 year)	217/957 (22.7%)	909/4352 (20.9%)	1.11 (0.94-1.31)	0.221	1.13 (0.95-1.34)	0.158	1.13 (0.95-1.34)	0.162
CP ≥2 (1 year)	99/957 (10.3%)	465/4352 (10.7%)	0.96 (0.77-1.21)	0.76	0.98 (0.78-1.23)	0.852	0.98 (0.78-1.24)	0.865
CP ≥3 (1 year)	48/957 (5.0%)	242/4352 (5.6%)	0.90 (0.65-1.23)	0.502	0.91 (0.66-1.25)	0.554	0.90 (0.66-1.24)	0.532
CP ≥1 (2 years)	346/957 (36.2%)	1,384/4352 (31.8%)	1.21 (1.05-1.41)	0.009*	1.25 (1.07-1.45)	0.004*	1.24 (1.07-1.44)	0.005*
Total cholesterol < 200mg/dL (n = 8242)								
CP ≥1 (1 year)	368/1685 (21.8%)	1,291/6557 (19.7%)	1.14 (1.00-1.30)	0.05	1.14 (1.00-1.31)	0.044*	1.14 (1.00-1.30)	0.05
CP ≥2 (1 year)	164/1685 (9.7%)	649/6557 (9.9%)	0.98 (0.82-1.18)	0.84	0.98 (0.82-1.18)	0.849	0.98 (0.82-1.18)	0.849
CP ≥3 (1 year)	83/1685 (4.9%)	358/6557 (5.5%)	0.90 (0.70-1.15)	0.385	0.90 (0.70-1.15)	0.402	0.89 (0.70-1.15)	0.378
CP ≥1 (2 years)	559/1685 (33.2%)	2010/6557 (30.7%)	1.12 (1.00-1.26)	0.046*	1.13 (1.01-1.27)	0.038*	1.13 (1.00-1.26)	0.044*
Total cholesterol ≥ 200mg/dL (n = 7518)								
CP ≥1 (1 year)	320/1467 (21.8%)	1170/6051 (19.3%)	1.16 (1.01-1.34)	0.033*	1.19 (1.03-1.37)	0.017*	1.19 (1.03-1.37)	0.017*
CP ≥2 (1 year)	150/1467 (10.2%)	567/6051 (9.4%)	1.10 (0.91-1.33)	0.318	1.13 (0.93-1.37)	0.209	1.13 (0.93-1.37)	0.219
CP ≥3 (1 year)	68/1467 (4.6%)	301/6051 (5.0%)	0.93 (0.71-1.22)	0.59	0.94 (0.72-1.23)	0.655	0.94 (0.71-1.23)	0.633
CP ≥1 (2 years)	508/1467 (34.6%)	1826/6051 (30.2%)	1.23 (1.09-1.38)	0.001*	1.26 (1.11-1.42)	<0.001*	1.25 (1.11-1.42)	<0.001*
CCI scores = 0 (n = 9601)								

CP ≥1 (1 year)	372/1687 (22.1%)	1573/7914 (19.9%)	1.14 (1.00-1.30)	0.044	1.16 (1.02-1.32)	0.028*	1.15 (1.01-1.31)	0.033*
CP ≥2 (1 year)	173/1687 (10.3%)	784/7914 (9.9%)	1.04 (0.87-1.24)	0.661	1.06 (0.89-1.26)	0.534	1.06 (0.89-1.26)	0.549
CP ≥3 (1 year)	91/1687 (5.4%)	417/7914 (5.3%)	1.03 (0.81-1.29)	0.834	1.04 (0.82-1.31)	0.751	1.04 (0.82-1.31)	0.769
CP ≥1 (2 years)	570/1687 (33.8%)	2446/7914 (30.9%)	1.14 (1.02-1.28)	0.021*	1.05 (0.80-1.37)	0.737	1.07 (0.81-1.39)	0.648
CCI score = 1 (n = 2599)								
CP ≥1 (1 year)	142/662 (21.5%)	361/1937 (18.6%)	1.19 (0.96-1.48)	0.114	1.22 (0.98-1.52)	0.078	1.23 (0.99-1.54)	0.068
CP ≥2 (1 year)	62/662 (9.4%)	174/1937 (9.0%)	1.05 (0.77-1.42)	0.765	1.06 (0.78-1.44)	0.704	1.07 (0.79-1.46)	0.67
CP ≥3 (1 year)	29/662 (4.4%)	97/1937 (5.0%)	0.87 (0.57-1.33)	0.517	0.89 (0.58-1.37)	0.594	0.87 (0.57-1.34)	0.539
CP ≥1 (2 years)	217/662 (32.8%)	588/1937 (30.4%)	1.12 (0.93-1.35)	0.245	0.71 (0.48-1.06)	0.096	0.72 (0.48-1.07)	0.101
CCI score ≥ 2 (n = 3,560)								
CP ≥1 (1 year)	174/803 (21.7%)	527/2757 (19.1%)	1.17 (0.97-1.42)	0.11	1.15 (0.95-1.40)	0.157	1.16 (0.95-1.41)	0.138
CP ≥2 (1 year)	79/803 (9.8%)	258/2757 (9.4%)	1.06 (0.81-1.38)	0.683	1.05 (0.80-1.37)	0.737	1.07 (0.81-1.39)	0.648
CP ≥3 (1 year)	31/803 (3.9%)	145/2757 (5.3%)	0.72 (0.49-1.08)	0.109	0.71 (0.48-1.06)	0.096	0.72 (0.48-1.07)	0.101
CP ≥1 (2 years)	280/803 (34.9%)	802/2757 (29.1%)	1.31 (1.10-1.54)	0.002*	1.29 (1.09-1.53)	0.003*	1.29 (1.09-1.52)	0.004*

CCI, Charlson Comorbidity Index; CP, chronic periodontitis; DBP, Diastolic blood pressure; SBP, Systolic blood pressure.

*Conditional or unconditional logistic regression analysis, significance at $P < 0.05$.

†Stratified model for age, sex, income, and geographic region.

‡Model 1 was adjusted for smoking status, alcohol use, obesity, and CCI scores.

§Model 2 was adjusted for model 1 plus total cholesterol, SBP, DBP, and fasting blood glucose.
