# Supplementary material for: Association of Chronic Periodontitis with Migraine in a Korean Adult Population: A Nationwide Nested Case-Control Study
Source: Healthcare (Basel). 2025 Aug 26;13(17):2123. doi: 10.3390/healthcare13172123 (PMC12428593; doi:10.3390/healthcare13172123)
Supplement: Supplementary file 1 [file healthcare-13-02123-s001.zip › Table S7 (Migraine without aura) - d.pdf]

Table S7. Subgroup analyses of crude and adjusted odds ratios according to age, sex, income, and region of residence

Characteristics	No. of case (exposure/total, %)	No. of control (exposure/total, %)	Odds ratios for migraine without aura (95% confidence interval)					
			Crude†	P-value	Model 1†‡	P-value	Model 2†§	P-value
Age < 60 years old (n = 89,900)								
CP ≥1 (1 year)	3623/17,980 (20.2%)	13,661/71,920 (19.0%)	1.08 (1.03-1.12)	<0.001*	1.08 (1.04-1.13)	<0.001*	1.09 (1.04-1.13)	<0.001*
CP ≥2 (1 year)	1689/17,980 (9.4%)	6544/71,920 (9.1%)	1.04 (0.98-1.10)	0.216	1.04 (0.99-1.10)	0.15	1.04 (0.99-1.11)	0.135
CP ≥3 (1 year)	930/17,980 (5.2%)	3657/71,920 (5.1%)	1.02 (0.95-1.10)	0.631	1.03 (0.95-1.11)	0.499	1.03 (0.95-1.11)	0.467
CP ≥1 (2 years)	5736/17,980 (31.9%)	21,575/71,920 (30.0%)	1.09 (1.06-1.13)	<0.001*	1.10 (1.06-1.14)	<0.001*	1.10 (1.07-1.15)	<0.001*
Age ≥ 60 years old (n = 111,135)								
CP ≥1 (1 year)	5475/22,227 (24.6%)	20,257/88,908 (22.8%)	1.11 (1.07-1.15)	<0.001*	1.11 (1.08-1.15)	<0.001*	1.11 (1.07-1.15)	<0.001*
CP ≥2 (1 year)	2713/22,227 (12.2%)	10,389/88,908 (11.7%)	1.05 (1.00-1.10)	0.031*	1.06 (1.01-1.10)	0.019*	1.05 (1.01-1.10)	0.023*
CP ≥3 (1 year)	1503/22,227 (6.8%)	5926/88,908 (6.7%)	1.02 (0.96-1.08)	0.604	1.02 (0.96-1.08)	0.489	1.02 (0.96-1.08)	0.522
CP ≥1 (2 years)	8274/22,227 (37.2%)	31,439/88,908 (35.4%)	1.08 (1.05-1.12)	<0.001*	1.09 (1.06-1.12)	<0.001*	1.09 (1.05-1.12)	<0.001*
Men (n = 68,780)								
CP ≥1 (1 year)	3600/13,756 (26.2%)	13,362/55,024 (24.3%)	1.11 (1.06-1.15)	<0.001*	1.11 (1.07-1.16)	<0.001*	1.11 (1.07-1.16)	<0.001*
CP ≥2 (1 year)	1793/13,756 (13.0%)	6906/55,024 (12.6%)	1.04 (0.99-1.10)	0.123	1.05 (0.99-1.11)	0.078	1.05 (1.00-1.11)	0.072
CP ≥3 (1 year)	1009/13,756 (7.3%)	3961/55,024 (7.2%)	1.02 (0.95-1.10)	0.579	1.03 (0.96-1.11)	0.442	1.03 (0.96-1.11)	0.422
CP ≥1 (2 years)	5490/13,756 (39.9%)	20,433/55,024 (37.1%)	1.12 (1.08-1.17)	<0.001*	1.13 (1.09-1.18)	<0.001*	1.13 (1.09-1.18)	<0.001*
Women (n = 132,255)								
CP ≥1 (1 year)	5498/26,451 (20.8%)	20,556/105,804 (19.4%)	1.09 (1.05-1.13)	<0.001*	1.09 (1.06-1.13)	<0.001*	1.09 (1.06-1.13)	<0.001*
CP ≥2 (1 year)	2609/26,451 (9.9%)	10,027/105,804 (9.5%)	1.05 (1.00-1.09)	0.053	1.05 (1.00-1.10)	0.037*	1.05 (1.00-1.10)	0.038*

CP ≥3 (1 year)	1424/26,451 (5.4%)	5622/105,804 (5.3%)	1.01 (0.96-1.08)	0.649	1.02 (0.96-1.08)	0.542	1.02 (0.96-1.08)	0.534
CP ≥1 (2 years)	8520/26,451 (32.2%)	32,581/105,804 (30.8%)	1.07 (1.04-1.10)	<0.001*	1.07 (1.04-1.10)	<0.001*	1.07 (1.04-1.10)	<0.001*
Low income (n = 98,170)								
CP ≥1 (1 year)	4211/19,634 (21.5%)	15,296/78,536 (19.5%)	1.13 (1.09-1.17)	<0.001*	1.14 (1.09-1.18)	<0.001*	1.13 (1.09-1.18)	<0.001*
CP ≥2 (1 year)	2026/19,634 (10.3%)	7474/78,536 (9.5%)	1.09 (1.04-1.15)	0.001*	1.10 (1.05-1.16)	<0.001*	1.10 (1.05-1.16)	<0.001*
CP ≥3 (1 year)	1124/19,634 (5.7%)	4217/78,536 (5.4%)	1.07 (1.00-1.15)	0.05	1.08 (1.01-1.15)	0.030*	1.08 (1.01-1.15)	0.030*
CP ≥1 (2 years)	6545/19,634 (33.3%)	24,250/78,536 (30.9%)	1.12 (1.08-1.16)	<0.001*	1.13 (1.09-1.16)	<0.001*	1.12 (1.09-1.16)	<0.001*
High income (n = 102,865)								
CP ≥1 (1 year)	4887/20,573 (23.8%)	18,622/82,292 (22.6%)	1.07 (1.03-1.10)	0.001*	1.07 (1.03-1.11)	<0.001*	1.07 (1.03-1.11)	<0.001*
CP ≥2 (1 year)	2376/20,573 (11.6%)	9459/82,292 (11.5%)	1.01 (0.96-1.05)	0.826	1.01 (0.96-1.06)	0.679	1.01 (0.96-1.06)	0.668
CP ≥3 (1 year)	1309/20,573 (6.4%)	5366/82,292 (6.5%)	0.97 (0.92-1.04)	0.414	0.98 (0.92-1.04)	0.517	0.98 (0.92-1.04)	0.534
CP ≥1 (2 years)	7465/20,573 (36.3%)	28,764/82,292 (35.0%)	1.06 (1.03-1.09)	<0.001*	1.07 (1.03-1.10)	<0.001*	1.07 (1.03-1.10)	<0.001*
Urban residents (n = 78,830)								
CP ≥1 (1 year)	4064/15,766 (25.8%)	14,737/63,064 (23.4%)	1.14 (1.09-1.19)	<0.001*	1.15 (1.10-1.19)	<0.001*	1.14 (1.10-1.19)	<0.001*
CP ≥2 (1 year)	1990/15,766 (12.6%)	7589/63,064 (12.0%)	1.06 (1.00-1.11)	0.043*	1.06 (1.01-1.12)	0.025*	1.06 (1.01-1.12)	0.031*
CP ≥3 (1 year)	1112/15,766 (7.1%)	4364/63,064 (6.9%)	1.02 (0.95-1.09)	0.554	1.03 (0.96-1.10)	0.424	1.03 (0.96-1.10)	0.458
CP ≥1 (2 years)	6081/15,766 (38.6%)	22,542/63,064 (35.7%)	1.13 (1.09-1.17)	<0.001*	1.14 (1.10-1.18)	<0.001*	1.13 (1.09-1.18)	<0.001*
Rural residents (n = 122,205)								
CP ≥1 (1 year)	5034/24,441 (20.6%)	19,181/97,764 (19.6%)	1.06 (1.03-1.10)	0.001*	1.07 (1.03-1.11)	<0.001*	1.07 (1.03-1.11)	<0.001*
CP ≥2 (1 year)	2412/24,441 (9.9%)	9344/97,764 (9.6%)	1.04 (0.99-1.09)	0.137	1.04 (0.99-1.09)	0.096	1.04 (0.99-1.09)	0.083

CP ≥3 (1 year)	1321/24,441 (5.4%)	5219/97,764 (5.3%)	1.01 (0.95-1.08)	0.679	1.02 (0.96-1.08)	0.555	1.02 (0.96-1.09)	0.518
CP ≥1 (2 years)	7929/24,441 (32.4%)	30,472/97,764 (31.2%)	1.06 (1.03-1.09)	<0.001*	1.07 (1.03-1.10)	<0.001*	1.07 (1.03-1.10)	<0.001*

CCI, Charlson Comorbidity Index; CP, chronic periodontitis; DBP, Diastolic blood pressure; SBP, Systolic blood pressure.

*Conditional or unconditional logistic regression analysis, significance at $P < 0.05$.

†Stratified model for age, sex, income, and geographic region.

‡Model 1 was adjusted for smoking status, alcohol use, obesity, and CCI scores.

§Model 2 was adjusted for model 1 plus total cholesterol, SBP, DBP, and fasting blood glucose.
