# Supplementary material for: Association of Chronic Periodontitis with Migraine in a Korean Adult Population: A Nationwide Nested Case-Control Study
Source: Healthcare (Basel). 2025 Aug 26;13(17):2123. doi: 10.3390/healthcare13172123 (PMC12428593; doi:10.3390/healthcare13172123)
Supplement: Supplementary file 1 [file healthcare-13-02123-s001.zip › Table S8 (Migraine without aura) - d.pdf]

Table S8. Subgroup analyses of crude and adjusted odds ratios according to obesity, smoking status, and alcohol consumption

Characteristics	No. of case (exposure/total, %)	No. of control (exposure/total, %)	Odds ratios for migraine with aura (95% confidence interval)					
			Crude†	P-value	Model 1†‡	P-value	Model 2†§	P-value
Underweight (n = 5077)								
CP ≥1 (1 year)	199/958 (20.8%)	711/4,119 (17.3%)	1.26 (1.05-1.50)	0.011*	1.26 (1.06-1.51)	0.010*	1.25 (1.05-1.50)	0.013*
CP ≥2 (1 year)	87/958 (9.1%)	343/4,119 (8.3%)	1.10 (0.86-1.41)	0.45	1.10 (0.86-1.41)	0.455	1.09 (0.85-1.40)	0.504
CP ≥3 (1 year)	43/958 (4.5%)	184/4,119 (4.5%)	1.01 (0.72-1.41)	0.977	1.00 (0.71-1.41)	0.989	1.00 (0.71-1.40)	0.983
CP ≥1 (2 years)	315/958 (32.9%)	1125/4,119 (27.3%)	1.30 (1.12-1.52)	0.001*	1.32 (1.13-1.53)	<0.001*	1.31 (1.13-1.53)	0.001*
Normal weight (n = 72,191)								
CP ≥1 (1 year)	3133/14,275 (22.0%)	11,868/57,916 (20.5%)	1.09 (1.04-1.14)	<0.001*	1.09 (1.04-1.14)	<0.001*	1.09 (1.04-1.14)	<0.001*
CP ≥2 (1 year)	1513/14,275 (10.6%)	5838/57,916 (10.1%)	1.06 (1.00-1.12)	0.066	1.06 (1.00-1.12)	0.075	1.06 (1.00-1.12)	0.073
CP ≥3 (1 year)	840/14,275 (5.9%)	3269/57,916 (5.6%)	1.05 (0.97-1.13)	0.262	1.04 (0.97-1.13)	0.278	1.05 (0.97-1.13)	0.275
CP ≥1 (2 years)	4809/14,275 (33.7%)	18,693/57,916 (32.3%)	1.07 (1.03-1.11)	0.001*	1.07 (1.03-1.11)	0.002*	1.07 (1.03-1.11)	0.001*
Overweight (n = 53,763)								
CP ≥1 (1 year)	2510/10,879 (23.1%)	9407/42,884 (21.9%)	1.07 (1.02-1.12)	0.011*	1.08 (1.02-1.13)	0.004*	1.08 (1.02-1.13)	0.005*
CP ≥2 (1 year)	1233/10,879 (11.3%)	4693/42,884 (10.9%)	1.04 (0.97-1.11)	0.241	1.05 (0.98-1.12)	0.167	1.05 (0.98-1.12)	0.172
CP ≥3 (1 year)	656/10,879 (6.0%)	2655/42,884 (6.2%)	0.97 (0.89-1.06)	0.535	0.98 (0.90-1.07)	0.676	0.98 (0.90-1.07)	0.649
CP ≥1 (2 years)	3893/10,879 (35.8%)	14,570/42,884 (34.0%)	1.08 (1.04-1.13)	<0.001*	1.09 (1.04-1.14)	<0.001*	1.09 (1.04-1.14)	<0.001*
Obese (n = 70,004)								
CP ≥1 (1 year)	3256/14,095 (23.1%)	11,932/55,909 (21.3%)	1.11 (1.06-1.16)	<0.001*	1.12 (1.07-1.17)	<0.001*	1.12 (1.07-1.17)	<0.001*
CP ≥2 (1 year)	1569/14,095 (11.1%)	6059/55,909 (10.8%)	1.03 (0.97-1.09)	0.313	1.04 (0.96-1.12)	0.369	1.04 (0.96-1.12)	0.341
CP ≥3 (1 year)	894/14,095 (6.3%)	3475/55,909 (6.2%)	1.02 (0.95-1.10)	0.575	1.04 (0.98-1.11)	0.157	1.04 (0.98-1.11)	0.151
CP ≥1 (2 years)	4993/14,095 (35.4%)	18,626/55,909 (33.3%)	1.10 (1.06-1.14)	<0.001*	1.11 (1.07-1.16)	<0.001*	1.11 (1.07-1.15)	<0.001*
Non-smoker (n = 158,152)								
CP ≥1 (1 year)	6931/31,959 (21.7%)	25,601/126,193 (20.3%)	1.09 (1.06-1.12)	<0.001*	1.09 (1.06-1.12)	<0.001*	1.09 (1.06-1.12)	<0.001*
CP ≥2 (1 year)	3324/31,959 (10.4%)	12,618/126,193 (10.0%)	1.05 (1.01-1.09)	0.024*	1.05 (1.01-1.09)	0.025*	1.04 (1.00-1.08)	0.051
CP ≥3 (1 year)	1822/31,959 (5.7%)	7074/126,193 (5.6%)	1.02 (0.97-1.08)	0.446	1.02 (0.97-1.08)	0.439	1.01 (0.96-1.06)	0.721
CP ≥1 (2 years)	10,655/31,959 (33.3%)	40,147/126,193 (31.8%)	1.08 (1.05-1.10)	<0.001*	1.07 (1.05-1.10)	<0.001*	1.08 (1.05-1.11)	<0.001*
Past and current smoker (n = 42,883)								
CP ≥1 (1 year)	2167/8,248 (26.3%)	8317/34,635 (24.0%)	1.13 (1.07-1.20)	<0.001*	1.13 (1.07-1.20)	<0.001*	1.12 (1.05-1.18)	<0.001*
CP ≥2 (1 year)	1078/8,248 (13.1%)	4315/34,635 (12.5%)	1.06 (0.99-1.14)	0.119	1.06 (0.99-1.14)	0.108	1.08 (1.00-1.16)	0.062
CP ≥3 (1 year)	611/8,248 (7.4%)	2509/34,635 (7.2%)	1.03 (0.94-1.13)	0.525	1.03 (0.94-1.13)	0.511	1.06 (0.96-1.16)	0.278
CP ≥1 (2 years)	3355/8,248 (40.7%)	12,867/34,635 (37.2%)	1.17 (1.11-1.23)	<0.001*	1.17 (1.11-1.23)	<0.001*	1.13 (1.07-1.19)	<0.001*
Alcohol consumption < 1 time a week (n = 161,270)								

CP ≥1 (1 year)	7189/32,699 (22.0%)	26,377/128,571 (20.5%)	1.09 (1.06-1.12)	<0.001*	1.09 (1.06-1.13)	<0.001*	1.09 (1.06-1.13)	<0.001*
CP ≥2 (1 year)	3459/32,699 (10.6%)	13,132/128,571 (10.2%)	1.04 (1.00-1.08)	0.051	1.04 (1.00-1.08)	0.046*	1.04 (1.00-1.08)	0.042*
CP ≥3 (1 year)	1894/32,699 (5.8%)	7381/128,571 (5.7%)	1.01 (0.96-1.06)	0.721	1.01 (0.96-1.07)	0.675	1.01 (0.96-1.07)	0.638
CP ≥1 (2 years)	11,102/32,699 (34.0%)	41,444/128,571 (32.2%)	1.08 (1.05-1.11)	<0.001*	1.08 (1.06-1.11)	<0.001*	1.08 (1.06-1.11)	<0.001*
Alcohol consumption ≥ 1 time a week (n = 39,765)								
CP ≥1 (1 year)	1909/7,508 (25.4%)	7541/32,257 (23.4%)	1.12 (1.05-1.18)	<0.001*	1.13 (1.06-1.19)	<0.001*	1.12 (1.06-1.19)	<0.001*
CP ≥2 (1 year)	943/7,508 (12.6%)	3801/32,257 (11.8%)	1.08 (1.00-1.16)	0.062	1.08 (1.00-1.17)	0.038*	1.08 (1.00-1.17)	0.040*
CP ≥3 (1 year)	539/7,508 (7.2%)	2202/32,257 (6.8%)	1.06 (0.96-1.16)	0.278	1.07 (0.97-1.18)	0.197	1.06 (0.96-1.17)	0.215
CP ≥1 (2 years)	2908/7,508 (38.7%)	11,570/32,257 (35.9%)	1.13 (1.07-1.19)	<0.001*	1.14 (1.09-1.20)	<0.001*	1.14 (1.08-1.20)	<0.001*

CCI, Charlson Comorbidity Index; CP, chronic periodontitis; DBP, Diastolic blood pressure; SBP, Systolic blood pressure.

*Conditional or unconditional logistic regression analysis, significance at $P < 0.05$.

†Stratified model for age, sex, income, and geographic region.

‡Model 1 was adjusted for smoking status, alcohol use, obesity, and CCI scores.

§Model 2 was adjusted for model 1 plus total cholesterol, SBP, DBP, and fasting blood glucose.
