# Supplementary material for: Association of Chronic Periodontitis with Migraine in a Korean Adult Population: A Nationwide Nested Case-Control Study
Source: Healthcare (Basel). 2025 Aug 26;13(17):2123. doi: 10.3390/healthcare13172123 (PMC12428593; doi:10.3390/healthcare13172123)
Supplement: Supplementary file 1 [file healthcare-13-02123-s001.zip › Table S9 (Migraine without aura) - d.pdf]

Table S9. Subgroup analyses of crude and adjusted odds ratios according to according to blood pressure, fasting blood glucose, total cholesterol, and CCI scores

Characteristics	No. of case	No. of control	Odds ratios for migraine without aura (95% confidence interval)					
	(exposure/total, %)	(exposure/total, %)	Crude†	P-value	Model 1†‡	P-value	Model 2†§	P-value
SBP < 140 mmHg and DBP < 90 mmHg (n = 56,848)								
CP ≥1 (1 year)	2715/11,660 (23.3%)	9983/45,188 (22.1%)	1.07 (1.02-1.12)	0.006*	1.07 (1.02-1.12)	0.009*	1.07 (1.02-1.12)	0.007*
CP ≥2 (1 year)	1303/11,660 (11.2%)	5009/45,188 (11.1%)	1.01 (0.95-1.08)	0.782	1.00 (0.94-1.07)	0.881	1.01 (0.95-1.08)	0.762
CP ≥3 (1 year)	729/11,660 (6.3%)	2874/45,188 (6.4%)	0.98 (0.90-1.07)	0.671	0.98 (0.90-1.06)	0.613	0.98 (0.90-1.07)	0.718
CP ≥1 (2 years)	4168/11,660 (35.8%)	15,475/45,188 (34.3%)	1.07 (1.02-1.11)	0.002*	1.07 (1.02-1.11)	0.004*	1.07 (1.02-1.12)	0.003*
SBP ≥ 140 mmHg or DBP ≥ 90 mmHg (n = 144,187)								
CP ≥1 (1 year)	6383/28,547 (22.4%)	23,935/115,640 (20.7%)	1.10 (1.07-1.14)	<0.001*	1.11 (1.08-1.15)	<0.001*	1.11 (1.08-1.15)	<0.001*
CP ≥2 (1 year)	3099/28,547 (10.9%)	11,924/115,640 (10.3%)	1.06 (1.02-1.10)	0.007*	1.07 (1.02-1.11)	0.002*	1.07 (1.02-1.11)	0.002*
CP ≥3 (1 year)	1704/28,547 (6.0%)	6709/115,640 (5.8%)	1.03 (0.98-1.09)	0.276	1.04 (0.98-1.10)	0.158	1.04 (0.98-1.10)	0.159
CP ≥1 (2 years)	9842/28,547 (34.5%)	37,539/115,640 (32.5%)	1.09 (1.07-1.13)	<0.001*	1.10 (1.07-1.14)	<0.001*	1.10 (1.07-1.13)	<0.001*
Fasting blood glucose < 100 mg/dL (n = 127,867)								
CP ≥1 (1 year)	5780/26,407 (21.9%)	20,405/101,460 (20.1%)	1.11 (1.08-1.15)	<0.001*	1.12 (1.08-1.15)	<0.001*	1.11 (1.08-1.15)	<0.001*
CP ≥2 (1 year)	2761/26,407 (10.5%)	9960/101,460 (9.8%)	1.07 (1.03-1.12)	0.002*	1.07 (1.03-1.12)	0.002*	1.07 (1.02-1.12)	0.003*
CP ≥3 (1 year)	1485/26,407 (5.6%)	5592/101,460 (5.5%)	1.02 (0.96-1.08)	0.476	1.02 (0.97-1.09)	0.429	1.02 (0.96-1.08)	0.523
CP ≥1 (2 years)	8887/26,407 (33.7%)	32,130/101,460 (31.7%)	1.09 (1.06-1.13)	<0.001*	1.10 (1.07-1.13)	<0.001*	1.09 (1.06-1.13)	<0.001*
Fasting blood glucose ≥ 100 mg/dL (n = 73,168)								
CP ≥1 (1 year)	3318/13,800 (24.0%)	13,513/59,368 (22.8%)	1.07 (1.03-1.12)	0.001*	1.08 (1.04-1.13)	<0.001*	1.08 (1.03-1.13)	0.001*
CP ≥2 (1 year)	1641/13,800 (11.9%)	6973/59,368 (11.8%)	1.01 (0.96-1.07)	0.631	1.02 (0.97-1.08)	0.448	1.02 (0.96-1.08)	0.593
CP ≥3 (1 year)	948/13,800 (6.9%)	3991/59,368 (6.7%)	1.02 (0.95-1.10)	0.533	1.03 (0.96-1.11)	0.388	1.03 (0.95-1.11)	0.478
CP ≥1 (2 years)	5123/13,800 (37.1%)	20,884/59,368 (35.2%)	1.09 (1.05-1.13)	<0.001*	1.10 (1.06-1.14)	<0.001*	1.09 (1.05-1.13)	<0.001*
Total cholesterol < 200mg/dL (n = 105,281)								
CP ≥1 (1 year)	4864/20,947 (23.2%)	18,060/84,334 (21.4%)	1.11 (1.07-1.15)	<0.001*	1.11 (1.07-1.16)	<0.001*	1.11 (1.07-1.15)	<0.001*
CP ≥2 (1 year)	2346/20,947 (11.2%)	9104/84,334 (10.8%)	1.04 (0.99-1.09)	0.089	1.05 (1.00-1.10)	0.072	1.04 (1.00-1.10)	0.077
CP ≥3 (1 year)	1291/20,947 (6.2%)	5144/84,334 (6.1%)	1.01 (0.95-1.08)	0.73	1.02 (0.95-1.08)	0.644	1.01 (0.95-1.08)	0.668
CP ≥1 (2 years)	7473/20,947 (35.7%)	28,055/84,334 (33.3%)	1.11 (1.08-1.15)	<0.001*	1.12 (1.08-1.15)	<0.001*	1.12 (1.08-1.15)	<0.001*
Total cholesterol ≥ 200mg/dL (n = 95,754)								

CP ≥1 (1 year)	4234/19,260 (22.0%)	15,858/76,494 (20.7%)	1.08 (1.04-1.12)	<0.001*	1.09 (1.04-1.13)	<0.001*	1.08 (1.04-1.13)	<0.001*
CP ≥2 (1 year)	2056/19,260 (10.7%)	7829/76,494 (10.2%)	1.05 (1.00-1.10)	0.07	1.06 (1.00-1.11)	0.038*	1.06 (1.00-1.11)	0.037*
CP ≥3 (1 year)	1142/19,260 (5.9%)	4439/76,494 (5.8%)	1.02 (0.96-1.09)	0.501	1.03 (0.96-1.10)	0.359	1.03 (0.97-1.11)	0.34
CP ≥1 (2 years)	6,537/19,260 (33.9%)	24,959/76,494 (32.6%)	1.06 (1.03-1.10)	0.001*	1.07 (1.03-1.11)	<0.001*	1.07 (1.03-1.10)	<0.001*
CCI scores = 0 (n = 120,280)								
CP ≥1 (1 year)	5163/22,261 (23.2%)	21,372/98,019 (21.8%)	1.08 (1.05-1.12)	<0.001*	1.09 (1.04-1.13)	<0.001*	1.10 (1.06-1.15)	<0.001*
CP ≥2 (1 year)	2533/22,261 (11.4%)	10,780/98,019 (11.0%)	1.04 (0.99-1.09)	0.099	1.09 (1.05-1.13)	<0.001*	1.11 (1.06-1.15)	<0.001*
CP ≥3 (1 year)	1411/22,261 (6.3%)	6189/98,019 (6.3%)	1.00 (0.95-1.07)	0.893	1.00 (0.94-1.06)	0.96	1.00 (0.94-1.06)	0.957
CP ≥1 (2 years)	7919/22,261 (35.6%)	33,063/98,019 (33.7%)	1.08 (1.05-1.12)	<0.001*	1.09 (1.04-1.13)	<0.001*	1.10 (1.06-1.15)	<0.001*
CCI score = 1 (n = 33,548)								
CP ≥1 (1 year)	837/7919 (10.6%)	2629/25,629 (10.3%)	1.11 (1.04-1.18)	0.001*	1.07 (0.99-1.14)	0.073	1.09 (1.02-1.17)	0.012*
CP ≥2 (1 year)	837/7919 (10.6%)	2629/25,629 (10.3%)	1.03 (0.95-1.12)	0.423	1.07 (1.00-1.15)	0.053	1.10 (1.03-1.18)	0.008*
CP ≥3 (1 year)	450/7919 (5.7%)	1456/25,629 (5.7%)	1.00 (0.90-1.12)	1	1.02 (0.91-1.13)	0.772	1.03 (0.92-1.15)	0.62
CP ≥1 (2 years)	2727/7919 (34.4%)	8412/25,629 (32.8%)	1.08 (1.02-1.13)	0.008*	1.07 (1.00-1.14)	0.069	1.10 (1.02-1.18)	0.011*
CCI score ≥2 (n = 47,207)								
CP ≥1 (1 year)	2168/10,027 (21.6%)	7261/37,180 (19.5%)	1.14 (1.08-1.20)	<0.001*	1.06 (1.00-1.12)	0.061	1.08 (1.02-1.15)	0.008*
CP ≥2 (1 year)	1032/10,027 (10.3%)	3524/37,180 (9.5%)	1.10 (1.02-1.18)	0.014*	1.06 (1.00-1.12)	0.05	1.09 (1.02-1.15)	0.006*
CP ≥3 (1 year)	572/10,027 (5.7%)	1938/37,180 (5.2%)	1.10 (1.00-1.21)	0.051	1.10 (1.00-1.21)	0.053	1.10 (1.00-1.21)	0.058
CP ≥1 (2 years)	3364/10,027 (33.6%)	11,539/37,180 (31.0%)	1.08 (1.02-1.13)	0.008*	1.06 (1.00-1.12)	0.065	1.08 (1.02-1.15)	0.009*

CCI, Charlson Comorbidity Index; CP, chronic periodontitis; DBP, Diastolic blood pressure; SBP, Systolic blood pressure.

*Conditional or unconditional logistic regression analysis, significance at P < 0.05.

†Stratified model for age, sex, income, and geographic region.

‡Model 1 was adjusted for smoking status, alcohol use, obesity, and CCI scores.

§Model 2 was adjusted for model 1 plus total cholesterol, SBP, DBP, and fasting blood glucose.
